# Effects of a Body-Based Mindfulness Program on Alexithymia, Dispositional Mindfulness, and Distress Symptoms: A Pilot Clinical Trial

**DOI:** 10.3390/bs15010055

**Published:** 2025-01-08

**Authors:** Rossella Mattea Quinto, Francesca Russo, Francesca Scafuto, Marco Innamorati, Federico Nitamo Montecucco, Silvia Ghiroldi

**Affiliations:** 1 Department of Human Sciences, European University of Rome, 00163 Rome, Italy; francesca.russo@unier.it (F.R.); marco.innamorati@unier.it (M.I.); 2 Department of Literature, Communication, Education and Society, University of Udine, 33100 Udine, Italy; 3 The Global Village NGO, Bagni di Lucca, 55022 Lucca, Italy; nitamo@yahoo.it (F.N.M.); silvia.ghiroldi@yahoo.it (S.G.)

**Keywords:** body-based mindfulness program, mind–body interventions, alexithymia, distress symptoms, dispositional mindfulness, healthy adults

## Abstract

Mind–body practices have shown to be effective in reducing psychological distress and improving well-being and quality of life in clinical samples and healthy populations. We tested the effects of a body-based mindfulness intervention based on alexithymia, distress symptoms, and dispositional mindfulness among healthy adults. A total of seventy-three participants (mean age 40.1) were assigned to a body-based mindfulness program (i.e., experimental group) and the control group (i.e., waiting list). Distress symptoms were assessed with the Symptoms Questionnaire; alexithymia was measured using the Toronto Alexithymia Scale; and dispositional mindfulness was assessed with the Five Facets Mindfulness Questionnaire. Measures were completed at baseline and at one-week post-test. Even when controlling for baseline score, the body-based mindfulness program has shown to be effective in reducing distress symptoms (i.e., anxiety, depression, and somatic symptoms) and alexithymia, as well as improving dispositional mindfulness (i.e., observing, acting with awareness, and non-reacting). The findings from this study provide evidence that the body-based mindfulness program may help individuals to integrate bodily expressions, thoughts, and emotions, keeping an integrated sense of self.

## 1. Introduction

Mind–body practices, including meditation and yoga, are part of complementary and alternative medicine and merge mental focus with active breathing and body movements in order to induce a general state of calm and inner peace (e.g., Walach et al., 2012). Although the term *mind–body practices* may be considered controversial (Lilienfeld et al., 2015), since it seems suggest a simplistic dualism between *mind* and *body*, it generally refers to a holistic approach (e.g., Greener, 2013). In this vein, these practices may be considered in an integrative perspective, wherein emotions, cognition, and physical aspects appear to be connected, in order to promote health and well-being. Mind–body practices have shown to be effective in reducing the psychological burden of chronic illnesses and contributing to the management of physical symptoms and psychological sequelae, and to successfully improve quality of life and psychological well-being in clinical samples (e.g., Casuso-Holgado et al., 2022; Younge et al., 2015; Ramirez-Garcia et al., 2019). However, little is known about the impact of these practices on mental health and emotional functioning in non-clinical populations.

Mind–body practices are mainly based on top-down and bottom-up processing. An important distinction should be made between bottom-up and top-down processes; specifically, the top-down process, which mainly occurs through the high-order processes of consciousness, needs willful attention on the bodily states, allowing the signals coming from the subcortical regions to be monitored and enhancing awareness and regulation of emotions; contrarily, the bottom-up process encompasses the influence of somatosensorial signals on high-order mental processes, including cognitive appraisal and emotional recognition (Taylor et al., 2010). Relevant examples of mind–body practices more based on top-down processing are the so-called *Mindfulness-Based Interventions* (MBIs). Specifically, mindfulness-based interventions (MBIs), defined as a specific and supportive intervention wherein participants are invited to pay attention in the present moment and non-judgmentally (Kabat-Zinn, 1994), have shown to be effective in reducing psychological distress, burnout, depression, and anxiety, as well as in promoting well-being and quality of life in non-clinical settings (Galante et al., 2021; Khoury et al., 2015). Moreover, mindful meditation includes exercises focusing on the body and/or breathing, leading to a broader awareness of internal states as well as an altered dynamic of consciousness (Sabe et al., 2019). Findings from a recent meta-analysis showed that meditation-based mind–body interventions may be an important and effective supplement to pharmacotherapy and psychotherapy in the treatment of a wide range of mental disorders (Vancampfort et al., 2021).

Recent findings paved the way for further research on the association between mindfulness, alexithymia, emotional regulation, and fear of connecting with such emotions (Lykins & Baer, 2009; Teixeira & Pereira, 2015). Specifically, alexithymia is described as a personality trait characterized by difficulties in recognizing and describing emotions, and an externally oriented style of thinking (i.e., the tendency of subjects to direct attention on external stimuli rather than internal thoughts and feelings; Bagby et al., 1994). Alexithymia has been found to be associated with somatization—even in the absence of somatic disease—in the general population (Mattila et al., 2008), and in adults with depression (Sagar et al., 2021), alcohol dependence (Honkalampi et al., 2022), eating disorders (Pace et al., 2023), and self-harm behaviors (Iskric et al., 2020). Furthermore, individuals high in dispositional mindfulness reported lower levels of alexithymia (e.g., Teixeira & Pereira, 2015).

Overall, alexithymia may hinder the success of any psychological intervention, although recent findings reported that it may be modified through specific therapeutic approaches (e.g., Cameron et al., 2014; Norman et al., 2019). Given that individuals high in alexithymia tend to fail in developing adequate mental representations of emotions, as the ability to differentiate and label emotions and the capacity to associate words to the physiological component of emotions are impaired, therapeutic approaches aiming to integrate the “perceptually-bound emotional experience” with the “conceptual affective representational level” may be effective (Taylor et al., 2024, p. 347). Moreover, techniques focusing on the ability to tolerate feelings and to increase awareness and imaginative capacity may be recommended, although improving awareness and emotional release can be overwhelming for patients with psychiatric disorders (e.g., Sheppes et al., 2011; Taylor et al., 2024). In this vein, according to the studies reviewed by Cameron et al. (2014), the most effective interventions for alexithymia generally use skills-based training designed to promote awareness of bodily sensations and the related emotions. Moreover, interventions focusing on improving alexithymia usually involve group therapy, and include exercises where the participants have to observe the way other people describe their feelings, experiences, and emotions (Cameron et al., 2014).

A vast and extensive body of literature has focused on the effects and mechanisms underlying the promotion of mental health and the reduction in psychological symptoms following mindfulness practice. It is now well established that mindfulness meditation can reduce levels of anxiety and depression in patients with various medical conditions (for systematic reviews, Bhattacharya & Hofmann, 2023; Hofmann et al., 2010), and among healthy adults (for a systematic review, de Vibe et al., 2017). Interestingly, many studies have focused on the effects of mindfulness meditation on somatic symptoms and somatization. In this context, Shaheen (2014) reviewed thirteen studies that had examined the effects of mindfulness-based therapy on somatic symptoms in patients with functional diseases, including fibromyalgia and irritable bowel syndrome. The review suggested that mindfulness could be effective in reducing pain, symptom severity, and depression, as well as in improving quality of life. However, in a recent study with a sample from the general population, Micheli et al. (2022) found that although most participants reported moderate to severe somatic symptoms, only about 30% practiced meditation. The same authors also found that participants with more expertise in mindfulness practice reported less somatic symptoms than practitioners with no experience.

Finally, dispositional mindfulness is one of the most frequently examined outcomes in the literature. Dispositional mindfulness is defined as a form of receptive awareness, characterized by the tendency to be mindful and to adopt a non-judgmental and accepting attitude (Brown & Ryan, 2003). Several meta-analyses focused on mindfulness-based intervention reported a moderate increase in dispositional mindfulness over the course of the mindfulness meditation (Quaglia et al., 2016; Sedlmeier et al., 2012; Visted et al., 2015), suggesting that training mindfulness through specific meditation sessions focused on enhancing awareness and present-moment experience can improve a relatively stable and dispositional skill. Quaglia et al. (2016) meta-analyzed fourteen studies to investigate which dimensions of dispositional mindfulness changes over time and whether specific moderators exist. The authors found that four of the five facets of dispositional mindfulness were more sensitive to a mindfulness-based intervention, with the effect size for the Describing dimension significantly lower than that of the other dimensions; moreover, the population treated (clinical vs. normative samples) was not a significant mediator of the effects of the training on dispositional mindfulness. Finally, significant associations between dispositional mindfulness and mental health outcomes emerged, suggesting the need for further investigations to understand the key processes underlying the relationship between the effects of a mindfulness training and the dispositional mindfulness of participants (Quaglia et al., 2016).

The general aim of this study was to examine whether a body-based mindfulness program may have a significant impact in reducing alexithymia and somatic complaints and in improving dispositional mindfulness in a sample of non-clinical adults. The program investigated here focused on the body–mind interaction and on the improvement of an integrative sense of self, stemming from other programs (Ghiroldi et al., 2020; Barbato & Montecucco, 2016). The idea to develop a body-oriented curriculum stemmed from the recognition that in mindfulness practice, mind or thought control is often highlighted, whereas the role of the body is often neglected (Tang et al., 2019). Moreover, each session addressed different themes, borrowed from a vast body of literature exploring embodied mindfulness, and combined top-down processing of meditation with bottom-up processing, starting from active exercises of body movement and breathing that stimulate involuntary attention to the body (e.g., Ogden et al., 2006; Khoury et al., 2017; Tang et al., 2019).

Specifically, we hypothesized that facets of alexithymia (i.e., difficulty in describing feelings, difficulty in identifying feelings, and externally oriented thinking), dispositional mindfulness (i.e., describing, observing, non-judging, non-reacting, and acting with awareness), and distress symptoms (i.e., depression, anxiety, anger/hostility, and somatic symptoms) may improve after a psychological intervention wherein participants are invited to experience mindful meditation associated with bodily exercises.

## 2. Materials and Methods

### 2.1. Sample

Seventy-three participants (mean age 40.1; SD = 9.7) were enrolled at the NGO Villaggio Globale (Bagni di Lucca, Italy) between February 2022 and March 2024 by two board-certified clinicians, specifically, a psychotherapist (S.G.) and a physician (F.N.M.). All patients agreed to participate in the study and signed an informed consent form in accordance with the Declaration of Helsinki. Eligibility criteria were age 18+ years, and the ability to read and speak Italian and to provide written informed consent. Exclusion criteria were the presence of a current mental disorder and having received psychotherapy and/or psychopharmacological treatment for at least 6 months in the last 3 years. The trial was conducted in accordance with the Consolidated Standards of Reporting Trials (CONSORT), and it was approved by the Ethics Committee for psychological research at the European University of Rome (Prot. N. 07/2024).

### 2.2. Measures

All the measures were completed by all the participants at the baseline and one week after completion of the intervention. The Symptoms Questionnaire (SQ; Fava et al., 1983; Kellner, 1987) was used to assess the distress symptoms. The SQ is a yes/no questionnaire with 92 brief and simple items; it contains four scales, i.e., anxiety, depression, somatic symptoms, and anger/hostility, and each scale contains two subscales: a symptom subscale (i.e., anxiety, depression, somatization, and anger/hostility) and a subscale of well-being (i.e., relaxation, contentment, physical well-being, and friendliness, respectively). In our sample, all scales showed good reliability (depression: pre-test α = 0.85, post-test α = 0.79; anxiety: pre-test α = 0.82, post-test α = 0.82; anger/hostility: pre-test α = 0.85, post-test α = 0.84; somatic symptoms: pre-test α = 0.82, post-test α = 0.80).

The Five-Facets Mindfulness Questionnaire (Bohlmeijer et al., 2011; Giovannini et al., 2014) was used to assess dispositional mindfulness; the instrument contains 24 items that are rated on a Likert-type scale ranging from 1 (never) to 5 (always) and five dimensions of dispositional mindfulness: describing (pre-test α = 0.90, post-test α = 0.92), observing (pre-test α = 0.80, post-test α = 0.79), non-judging (pre-test α = 0.91, post-test α = 0.91), non-reacting (pre-test α = 0.89, post-test α = 0.89), and acting with awareness (pre-test α = 0.91, post-test α = 0.94).

The Toronto Alexithymia Scale (TAS-20; Bagby et al., 1994; Bressi et al., 1996) was used to measure alexithymia. It contains 20 items rated on a 5-point Likert-type scale (from 1, strongly disagree, to 5, strongly agree). The TAS-20 measures three dimensions of alexithymia: (1) difficulty in identifying feelings (DIF; pre-test α = 0.87, post-test α = 0.86); (2) difficulty in describing feelings (DDF; pre-test α = 0.80, post-test α = 0.78); and (3) externally oriented thinking (EOT; pre-test α = 0.53, post-test α = 0.50). Due to the poor reliability, the EOT dimension of TAS was not included in the analyses.

### 2.3. Procedure

The present pilot clinical trial compared outcomes for participants assigned to a body-based mindfulness program (i.e., experimental group) and a control group. The experimental group included 55 participants (about 75% of the sample), while 18 participants in the control group (about 25% of the sample) were allocated to a waiting list condition (a flowchart of participants is provided in Figure S1). Participants in the experimental group were recruited at their enrollment to the first-year basic course group activities, while participants in the control group were invited to participate in the study during open day events organized by the NGO Villaggio Globale. Participants in the control group were not involved in any research or intervention activities at the NGO Villaggio Globale.

The assignment to groups was not random. After obtaining informed consent, all participants received information about the aims and activities of the study. Participants in the intervention group were invited to complete the questionnaires in paper format. The first evaluation took place during the first day of the course, while the post-test was conducted one week after the conclusion of the intervention. For participants in the control group, only the first administration of the questionnaire was conducted in person. To ensure anonymity, all participants generated an alphanumeric code and provided access to personal contact information (e.g., phone number or e-mail address). Participants in the waiting list group were contacted via phone or e-mail and completed the second evaluation using Google Forms. Before completing the assessment protocol with Google Forms, all participants were informed of their rights, according to the EU Regulation no. 2016/679 and Italian Personal Data Protection Code (D.Lgs. no. 196/2003), and about emergency telephone numbers.

Three mindfulness instructors, who had more than three years of meditation experience as well as psychotherapeutic competence, were engaged in an intensive training to learn to conduct the body-based mindfulness program, wherein they were instructed on the aims and the activities of each intervention session. In the present clinical trial, blinding of both outcome assessor and participants to the content of the mindfulness intervention was not possible.

The Body-Based Mindfulness Program. The program included 9 residential sessions (once a month); each session consisted of four activities: (1) the operators explain the most important theme to be examined in the session to the participants; (2) meditation exercises are performed and participants are invited to bring their attention to internal experiences, in particular physical ones, and to explore interoceptive sensitivity; (3) participants are instructed to carry out specific mind–body exercises, namely breathing and emotional–bodily exercises, role playing, and acting out, aimed at allowing participants to explore their own difficult past experiences and facilitating physical catharsis, expression, or release of emotions; and (4) participants learn to share their feelings, in pairs or in group of 4 participants or more, and to live in the present moment and experience self-integration.

In the first stage of the training (i.e., first session), participants are instructed on the general aims of the program, the group setting methods, and the meditation practices; the instructors explore the motivations and expectations of each participant. The second and third sessions focus on the self-disclosure of each participant about difficult past experiences, including neglect or mistreatment and invalidation of emotions, and involving primary figures (i.e., parents), considering that the relationship with parents during childhood could be associated strongly with difficulties in accessing and managing emotional experiences, as well as with disturbances in psycho-affective development (e.g., Schimmenti & Caretti, 2018; Zhang et al., 2024). The fourth session focuses on emotional, bodily, and cognitive experiences associated with possible traumatic events, re-experiencing the memories that are associated with what they perceive as psychological and physical blockages; the aim of the exercises is to promote self-integration (e.g., Sapriel, 2012). In the fifth one, participants are involved in an intensive meditation session aimed at improving self-integrity and interoception, as well as developing more awareness and a non-judging attitude towards traumatic events (e.g., Barbato & Montecucco, 2016). The sixth session focuses on one’s beliefs about oneself and on how these interfere with one’s personal power, assertiveness, and self-determination, while the seventh and eighth sessions focus on the promotion of psychological well-being, in its eudaimonic components, working on the awareness about one’s self-integrity and authentic self-determination (Ryff & Singer, 2008). In particular, through mindfulness meditation, participants can learn how to improve positive characteristics of well-being, like personal growth and purpose in life (Scafuto et al., 2024a). In the final session, participants are invited to be re-exposed and re-experience the more difficult memories of their life, to find broader meaning in life and explore what they can learn from those (Allan et al., 2015; Tedeschi & Blevins, 2015). The objectives and activities of each intervention session are detailed in Table S1.

### 2.4. Statistical Analysis

In a post hoc power analysis conducted using G*Power, the statistical power for detecting the effect in the current study was found to be 0.90 (α = 0.05; effect size *f*^2^(V) = 0.38; sample size = 73; number of groups = 2; number of measurements = 2), which exceeds the commonly accepted threshold of 0.80, indicating that the study had adequate power to detect the effect. All statistical analyses were performed using SPSS (Version 27.0; IBM Corp., Armonk, NY, USA). All data were checked for normality. Given that all variables showed skewness and kurtosis lower than 1, we used parametric testing.

A series of *t*-tests and chi-squared tests were used to determine whether the two groups were equivalent at the baseline for demographic and outcome variables. Pre–post Cohen’s *d* was used to assess effect sizes. We conducted a generalized linear mixed model with fixed and random effects to determine whether the body-based mindfulness program was effective in increasing positive psychological factors (i.e., dispositional mindfulness dimensions, namely observing, describing, acting with awareness, non-judging, and non-reacting) as well as in decreasing negative psychological factors (i.e., alexithymia and somatic complaints). For all the variables we tested, the same mixed model was used, which included the fixed effects of treatment (control vs. treatment), time (baseline vs. post-test), and their interaction (treatment by time), and age as the covariate. A significant interaction effect would indicate a significant effect of the training. To consider the effect of dependence between temporal measures, the subjects’ id was included as a random effect, allowing variation in the intercept between individuals.

All statistics were considered significant at *p* < 0.05. Occasional missing values were imputed by calculating the mean score of the subscale for each participant, and then replaced.

## 3. Results

Table 1 lists means and standard deviations for outcome measures at the baseline and post-test broken down by group. Participants in the experimental group (compared to controls) reported higher levels of difficulty in identifying (t = 2.24; *p* = 0.031) and describing feelings (t = 2.65; *p* = 0.011), as well as lower levels of describing, as measured with the FFMQ (t = −2.06; *p* = 0.049), and higher hostility (t = −2.63; *p* = 0.012) and anxiety (t = −2.11; *p* = 0.043) at the baseline. Groups did not differ significantly on other outcome variables at the baseline. Moreover, although the groups did not differ for sex, controls were older than participants in the experimental group.

### Treatment Effects

The effect of the body-based mindfulness intervention over time was significant (*p* < 0.05) for most dimensions of dispositional mindfulness, alexithymia, and somatic complaints. For dispositional mindfulness (Table 2), we found a significant treatment-by-time interaction for the majority of facets, including observing (B = 3.35; SE = 1.14; *p* = 0.004), describing (B = 2.89; SE = 1.15; *p* = 0.014), acting with awareness (B = 3.09; SE = 1.28; *p* = 0.018), and non-reacting (B = 2.68; SE = 1.04; *p* = 0.012). For alexithymia (Table 3), we found a significant effect for difficulty in identifying (B = −2.85; SE = 1.22; *p* = 0.022) and describing feelings (B = −3.22; SE = 0.95; *p* = 0.001), as well as the overall TAS-20 score (B = −6.68; SE = 2.37; *p* = 0.006). Finally, for psychological symptoms (Table 4), significant treatment-by-time interactions emerged in all facets, namely anxiety (B = −4.35; SE = 0.94; *p* < 0.001), depression (B = −0.98; SE = 0.45; *p* = 0.032), somatic symptoms (B = −4.10; SE = 1.10; *p* < 0.001), and hostility (B = −4.22; SE = 0.99; *p* < 0.001).

## 4. Discussion

This study has examined the effect of a body-based mindfulness program on alexithymia, dispositional mindfulness, and psychological symptoms in a sample of non-clinical adults. Compared to the controls, participants in the training group reported reduced levels of anxiety, depression, hostility, and somatic symptoms, as well as alexithymia. Moreover, our program has shown to be effective for improving most dispositional mindfulness facets. However, it is worth noting that given the statistically significant between-group differences in several outcome dimensions (i.e., both dimensions of alexithymia, the describing dimension as measured with FFMQ, and the hostility dimension as assessed with SQ) at baseline, we must cautiously interpret our results regarding those dimensions in relation to the effectiveness of the program.

Previous studies reported similar results. In particular, Bornemann and Singer (2017) tested the effectiveness of a mindfulness-based intervention on alexithymia and heartbeat perception accuracy in a group of healthy adults. Their intervention consisted of three modules: the Presence module, wherein participants were instructed to direct their attention to the present moment; the Affect module, in which participants were familiarized with specific methods to regulate and accept difficult emotions as well as to increase prosocial attitudes; and the Perspective module, which is focused on metacognition and on observing thoughts and reframing experiences. The authors of the study found that participants in the experimental groups had lower levels of alexithymia at the six- and nine-month follow-up compared to the controls (Bornemann & Singer, 2017). Moreover, as pointed out by the same authors, changes in alexithymia were associated with the Presence and the Affect modules, but not with the Perspective module, suggesting that learning to read bodily signals through contemplative training could influence the ability to understand one’s emotions (Bornemann & Singer, 2017). However, although they used breathing meditation and body scans, they did not work on either traumatic past experiences or on eudaimonic well-being for developing feelings of personal power.

In one other study with healthy adults, a mindfulness-based stress reduction program was shown to be effective in reducing alexithymia, as well as worry, anxiety state, and depression (Santarnecchi et al., 2014). These changes were observed immediately after the intervention in the experimental group, but not in the control group (Santarnecchi et al., 2014). Moreover, Viding et al. (2015) tested the effect of an intervention consisting of six cultural activities, and including mindfulness training focused on breathing, body awareness, and awareness of thoughts and feelings; participants in the experimental group reported a significantly greater decrease in alexithymia and difficulty in identifying and describing feelings at the 6-month follow-up compared to controls. The authors suggested that exploring bodily sensations and enhancing awareness of physical correlates of emotions may reduce the difficulties in experiencing and describing feelings (Viding et al., 2015). Finally, D’Antoni et al. (2022) found that dispositional mindfulness, as well as interoceptive awareness, increased through a mindfulness meditation program; moreover, the authors reported that the mindfulness meditation was effective in significantly reducing dissociative symptoms (D’Antoni et al., 2022).

Overall, our study added knowledge about the key mechanisms through which a body-based mindfulness program can affect emotional mentalization, psychological symptoms, and dispositional mindfulness. According to the procedure of our therapeutic sessions, the two key elements for improving our psychological outcomes are self-integrity and interoceptive awareness. On one hand, interoception, defined as “an iterative process, requiring the interplay between perception of body states and cognitive appraisal of these states to inform response selection” (Farb et al., 2015, p. 2), might have been crucial in improving our outcomes, particularly alexithymia. On the other hand, self-integrity, defined as “a process and tendency that individuals build connections between differentiating and multiplying self-components, increase the level of conceptualization, and eventually form a sustainable and stable core self” (Zhou et al., 2022, p. 464), may have enabled a general improvement in mental health in our sample, with particular attention to the reduction in somatic symptoms.

The importance of the concept of interoception for understanding the mechanisms through which mindfulness can impact mental health and emotional functioning has already been investigated (e.g., Silveira et al., 2023; Gibson, 2019). In particular, alexithymia has even been defined as a marker of atypical interoception (Shalev, 2019), suggesting that the failure to process emotions may be derived from a misinterpretation of bodily sensation and difficulty in interoceptive awareness. In this vein, Luminet and Nielson (2024) argued that the difficulty in identifying feelings (DIF dimension of TAS-20) “includes difficulty distinguishing one’s feelings from internal bodily sensations and states, known as interoception” (p. 6). However, the definition of interoceptive awareness as the perception of the body’s internal state encompasses not only sensory awareness but also subjective appraisal, attitudes, beliefs, past experiences, personal expectations, and contextual influences (e.g., Craig, 2002; Farb et al., 2015), providing, with all these elements integrated, a moment-by-moment representation of one’s bodily landscape and a more mindful self-concept (Gibson, 2019).

In line with these approaches, it can be argued that working on interoception might improve alexithymia, even in light of the possible overlaps often examined in neuroscientific studies (e.g., Ernst et al., 2014) between dispositional mindfulness and psychological symptoms, especially somatic complaints. As pointed out by Farb et al. (2015), mind–body therapies, including mindfulness, could promote changes in interoceptive processing by enhancing the bottom-up integration of bodily sensations rather than attempting to alter them to fit top-down expectations. Interoception, enhanced by mindfulness or contemplative mind–body practices, could be considered as a cognitive appraisal of body states through which experiencing more insight into inner bodily sensations may, in turn, make a significant reduction in overall psychological symptoms possible. Thus, it is likely that our body-based mindfulness program could have led participants to be more aware of the nature of their body sensations, even those associated with negative emotions, and induced the reduction in the somatic symptoms experienced and enhanced insight and broader attention.

Finally, self-integrity has been crucial in our intervention as it worked with traumatic past memories. In this context, participants were taught mindful breathing and body scanning techniques to regulate autonomic nervous system activation, helping them achieve an optimal level of arousal to process traumatic memories and emotions effectively, without resorting to suppression, dissociation, or becoming overwhelmed (e.g., Kelly & Garland, 2016). Focusing on the present moment and experiencing past traumatic memories in a non-judgmental way may have led participants in the experimental group to integrate the present and the past, as well as their thoughts, feelings, and somatic sensations.

In sum, a pointed out in our previous studies that showed the positive effects of a body-based mindfulness program adapted to children and adolescents for emotional regulation, well-being, and internalizing and externalizing problems (Ghiroldi et al., 2020; Scafuto et al., 2022, 2024a, 2024b), the body–mind interaction enhanced through mindfulness meditation may be an important approach to increase self-integrity and the awareness of one’s psychological state starting from proprioceptive and interoceptive exercises. Indeed, the positive effect on alexithymia may stem from particular characteristics of our program, which intervene by working on the integration of physical correlates of emotions, past traumatic experiences, and current physiological states and tendencies. These mechanisms may lead to a more integrative self, merging physical sensations, cognitive experience of feelings, and emotional patterns of traumatic memories, and thus leading people to be more able to identify and describe their emotions and to experience lower levels of psychological symptoms.

### Limitations and Directions for Future Research

This study has some limitations. First, as we enrolled participants in the experimental group from a course group, the assignment to the experimental and control groups was neither random nor blinded; moreover, we found statistically significant between-group differences at the baseline for important variables, which led us to interpret our results cautiously. Second, participants in the control group were fewer than those in the experimental group due to the difficulties associated with recruiting participants from a waiting list. Third, we did not include an active control group to avoid the Hawthorne effect. Fourth, we controlled only for age, and we did not measure other potential confounding variables. Moreover, our variables of interest were assessed only by self-report measures, which are potentially biased by social desirability. Finally, this study may have overemphasized the short-term effects as it did not include a follow-up assessment to ascertain the stability of the effects over time.

In light of the limitations cited above, future studies should expand the sample size to achieve greater statistical power. Given the high incidence of our outcome measures within the clinical population, it would be important to test the effectiveness of our intervention in patients with chronic or degenerative medical conditions. Moreover, future research should include an active control group to better identify and detect the psychological mechanisms underlying the associations between the effects of the body-based mindfulness intervention and the measured outcomes, i.e., alexithymia, psychological symptoms, and dispositional mindfulness. It is also crucial to control for other potential confounding variables (such as the number of recent/past traumatic events) to enhance the conclusions regarding the effectiveness of the intervention. Finally, the use of psychophysiological or neuroimaging measures would be desirable to complement findings from self-report data and provide greater insight into the brain pathways activated by the body-based mindfulness intervention.

## 5. Conclusions

The current study provides important evidence that a body-based mindfulness program could be effective in reducing alexithymia and somatic complaints and in improving dispositional mindfulness in healthy adults. We found that mindfulness meditation, associated with body-based practices and training focused on interoception, self-integrity, and emotional awareness, could be effective in enhancing mentalization of emotions and in promoting a better understanding of one’s bodily sensations. Our findings also suggest that alexithymia can be considered changeable and that focusing on the body may help individuals to integrate their bodily expressions, thoughts, and emotions, keeping their sense of self. Finally, considering the aims of the body-based mindfulness program, future studies should test its effects on other health outcomes, including bodyfulness and emotion regulation.

## Figures and Tables

**Table 1:** Means and standard deviations (in parentheses) for outcome measures in the experimental and control groups at the baseline and post-test.

Outcome Measures	Control Group (*n* = 18)			Treatment Group (*n* = 55)			
	Baseline	Post-Test	Cohen’s *d* Effect Size ^a^	Baseline	Post-Test	Cohen’s *d* Effect Size ^a^	*p*-Value ^b^
FFMQ Observing	29.28 (4.2)	27.78 (4.1)	3.61	28.71 (5.7)	30.56 (4.7)	3.54	0.651
FFMQ Describing	31.44 (6.4)	31.56 (6.3)	0.19	27.87 (6.2)	30.87 (5.5)	5.12	0.049
FFMQ Acting with Awareness	28.00 (7.1)	27.06 (8.0)	1.24	25.71 (6.3)	27.85 (5.5)	3.62	0.232
FFMQ Non-judging	29.44 (9.2)	31.06 (7.1)	1.97	26.91 (6.9)	31.20 (6.2)	6.54	0.296
FFMQ Non-reacting	21.33 (5.6)	21.78 (5.0)	0.85	19.35 (5.5)	22.47 (5.2)	5.83	0.200
TAS-20 DIF	14.72 (5.5)	14.56 (6.3)	0.27	18.36 (7.2)	15.35 (6.2)	4.48	0.031
TAS-20 DDF	9.44 (3.3)	10.72 (3.9)	3.54	12.15 (5.0)	10.20 (4.4)	4.15	0.011
TAS-20 Total score	40.22 (7.7)	40.22 (7.8)	0.00	45.62 (13.2)	40.98 (11.0)	3.82	0.038
SQ Anxiety	6.33 (4.6)	6.67 (4.9)	0.72	9.02 (5.1)	5.00 (4.1)	8.69	0.043
SQ Depression	5.61 (5.5)	5.61 (5.7)	0.00	4.78 (3.7)	3.80 (3.3)	2.80	0.559
SQ Somatic symptoms	7.89 (4.6)	8.44 (4.6)	1.20	9.51 (5.6)	5.96 (4.8)	6.81	0.229
SQ Hostility	4.06 (3.9)	5.11 (4.3)	2.56	7.07 (5.2)	3.91 (3.9)	6.88	0.012

*Note*. Cohen’s *d* effect size ^a^ = within-group Cohen’s *d* effect size; *p*-value ^b^ = comparison of baseline values between intervention and control group; with *t*-test FFMQ = Five Facets Mindfulness Questionnaire; TAS-20 DIF = Toronto Alexithymia Scale-20—Difficulty in Identifying Feelings; TAS-20 DIF = Toronto Alexithymia Scale-20—Difficulty in Describing Feelings; SQ = Symptom Questionnaire.

**Table 2:** Effects of the body-based mindfulness intervention on facets of dispositional mindfulness (i.e., observing, describing, acting with awareness, non-judging, and non-reacting).

		Estimate	*SE*	*t*	*p*-Test	95% CI	
						LL	UL
Observing							
	Constant	29.09	2.31	12.59	<0.001	24.52	33.67
	Treatment	−2.83	1.44	−1.97	0.051	−5.66	0.01
	Time	−2.19	0.66	−3.30	0.001	−3.50	−0.88
	Age	0.03	0.06	0.60	0.547	−0.08	0.15
	Treatment-by-time interaction	3.69	1.33	2.77	0.006	1.06	6.31
Describing							
	Constant	24.79	2.75	9.00	<0.001	19.34	30.23
	Treatment	−0.02	1.63	−0.01	0.990	−3.24	3.20
	Time	−3.02	0.57	−5.25	<0.001	−4.16	−1.88
	Age	0.15	0.07	2.27	0.025	0.02	0.29
	Treatment-by-time interaction	2.91	1.15	2.52	0.013	0.63	5.19
Acting with Awareness							
	Constant	24.44	2.98	8.21	<0.001	18.56	30.33
	Treatment	−1.17	1.76	−0.66	0.508	−4.66	2.32
	Time	−2.21	0.64	−3.46	<0.001	−3.48	−0.95
	Age	0.09	0.07	1.17	0.244	−0.06	0.23
	Treatment-by-time interaction	3.16	1.28	2.46	0.015	0.63	5.69
Non judging							
	Constant	29.15	3.17	9.19	<0.001	22.89	35.42
	Treatment	−0.28	1.96	−0.15	0.885	−4.16	3.59
	Time	−4.18	0.89	−4.72	<0.001	−5.94	−2.43
	Age	0.05	0.08	0.64	0.525	−0.11	0.20
	Treatment-by-time interaction	2.57	1.78	1.45	0.150	−0.94	6.09
Non reacting							
	Constant	21.66	2.53	8.56	<0.001	16.66	26.66
	Treatment	−0.78	1.50	−0.53	0.601	−3.73	2.17
	Time	−3.13	0.52	−6.01	<0.001	−4.16	−2.10
	Age	0.02	0.06	0.33	0.743	−0.10	0.14
	Treatment-by-time interaction	2.69	1.04	2.57	0.011	0.62	4.75

*Note*. 95% CI = 95% confidence interval; LL = lower level; UL = upper level.

**Table 3:** Effects of the body-based mindfulness intervention on facets of alexithymia (i.e., difficulty in identifying feelings, difficulty in describing feelings, externally oriented thinking).

		Estimate	*SE*	*t*	*p*-Test	95% CI	
						LL	UL
Difficulty in Identifying Feelings (TAS-20 DIF)							
	Constant	16.99	3.11	5.46	<0.001	10.84	23.14
	Treatment	−0.68	1.83	−0.37	0.709	−4.30	2.93
	Time	3.05	0.61	4.97	<0.001	1.84	4.27
	Age	−0.04	0.08	−0.52	0.605	−0.19	0.11
	Treatment-by-time interaction	−2.88	1.23	−2.35	0.020	−5.32	−0.45
Difficulty in Describing Feelings (TAS-20 DDF)							
	Constant	13.49	2.05	6.57	<0.001	9.43	17.55
	Treatment	0.93	1.23	0.76	0.452	−1.50	3.36
	Time	2.01	0.47	4.25	<0.001	1.08	2.95
	Age	−0.08	0.05	−1.66	0.099	−0.18	0.02
	Treatment-by-time interaction	−3.29	0.95	−3.46	<0.001	−5.17	−1.41
TAS-20 Total Score							
	Constant	46.63	5.26	8.87	<0.001	36.23	57.03
	Treatment	1.73	3.14	0.55	0.583	−4.48	7.93
	Time	4.68	1.18	3.96	<0.001	2.34	7.02
	Age	−0.14	0.13	−1.07	0.285	−0.40	0.12
	Treatment-by-time interaction	−6.68	2.37	−2.82	0.006	−11.36	−1.99

*Note*. 95% CI = 95% confidence interval; LL = lower level; UL = upper level.

**Table 4:** Effects of the body-based mindfulness intervention on subscales of the Symptom Questionnaire Score (i.e., anxiety, depression, somatization, and hostility).

		Estimate	*SE*	*t*	*p*-Test	95% CI	
						LL	UL
Anxiety							
	Constant	10.24	2.10	4.88	<0.001	6.09	14.38
	Treatment	2.42	1.25	1.93	0.055	−0.06	4.90
	Time	3.93	0.48	8.20	<0.001	2.98	4.87
	Age	−0.14	0.05	−2.63	0.009	−0.24	−0.03
	Treatment-by-time interaction	−4.26	0.95	−4.44	<0.001	−6.16	−2.36
Depression							
	Constant	6.14	2.01	3.06	0.003	2.17	10.11
	Treatment	2.19	1.14	1.93	0.056	−0.05	4.44
	Time	0.99	0.22	4.44	<0.001	0.55	1.44
	Age	−0.06	0.05	−1.24	0.216	−0.16	0.04
	Treatment-by-time interaction	−0.99	0.45	−2.22	0.028	−1.89	−0.11
Somatic Symptoms							
	Constant	9.86	2.33	4.23	<0.001	5.25	14.17
	Treatment	2.92	1.39	2.10	0.037	0.17	5.67
	Time	3.40	0.52	6.52	<0.001	2.37	4.43
	Age	−0.10	0.06	−1.71	0.089	−0.21	0.02
	Treatment-by-time interaction	−3.95	1.04	−3.79	<0.001	−6.02	−1.89
Hostility							
	Constant	9.63	1.95	4.92	<0.001	5.76	13.49
	Treatment	2.00	1.19	1.69	0.094	−0.34	4.35
	Time	3.15	0.50	6.36	<0.001	2.17	4.13
	Age	−0.15	0.05	−3.08	0.003	−0.24	−0.05
	Treatment-by-time interaction	−4.21	0.99	−4.23	<0.001	−6.18	−2.24

*Note*. 95% CI = 95% confidence interval; LL = lower level; UL = upper level.

## Data Availability

The raw data supporting the conclusions of this article will be made available by the authors on request.

## Supplementary Materials

The following supporting information can be downloaded at: https://www.mdpi.com/article/10.3390/bs15010055/s1, Figure S1. Flowchart of participants. Table S1. The Mindfulness Body-based Intervention: procedure, aims, and target effects.
