# Supplementary material for: Effects of a Body-Based Mindfulness Program on Alexithymia, Dispositional Mindfulness, and Distress Symptoms: A Pilot Clinical Trial
Source: Behav Sci (Basel). 2025 Jan 8;15(1):55. doi: 10.3390/bs15010055 (PMC11763314; doi:10.3390/bs15010055)
Supplement: Supplementary file 1 [file behavsci-15-00055-s001.zip › behavsci-3356514-supplementary.pdf]

behavioral sciences

MDPI

Effects of a Body-Based Mindfulness Program on Alexithymia, Dispositional Mindfulness, and Distress Symptoms: A Pilot Clinical Trial

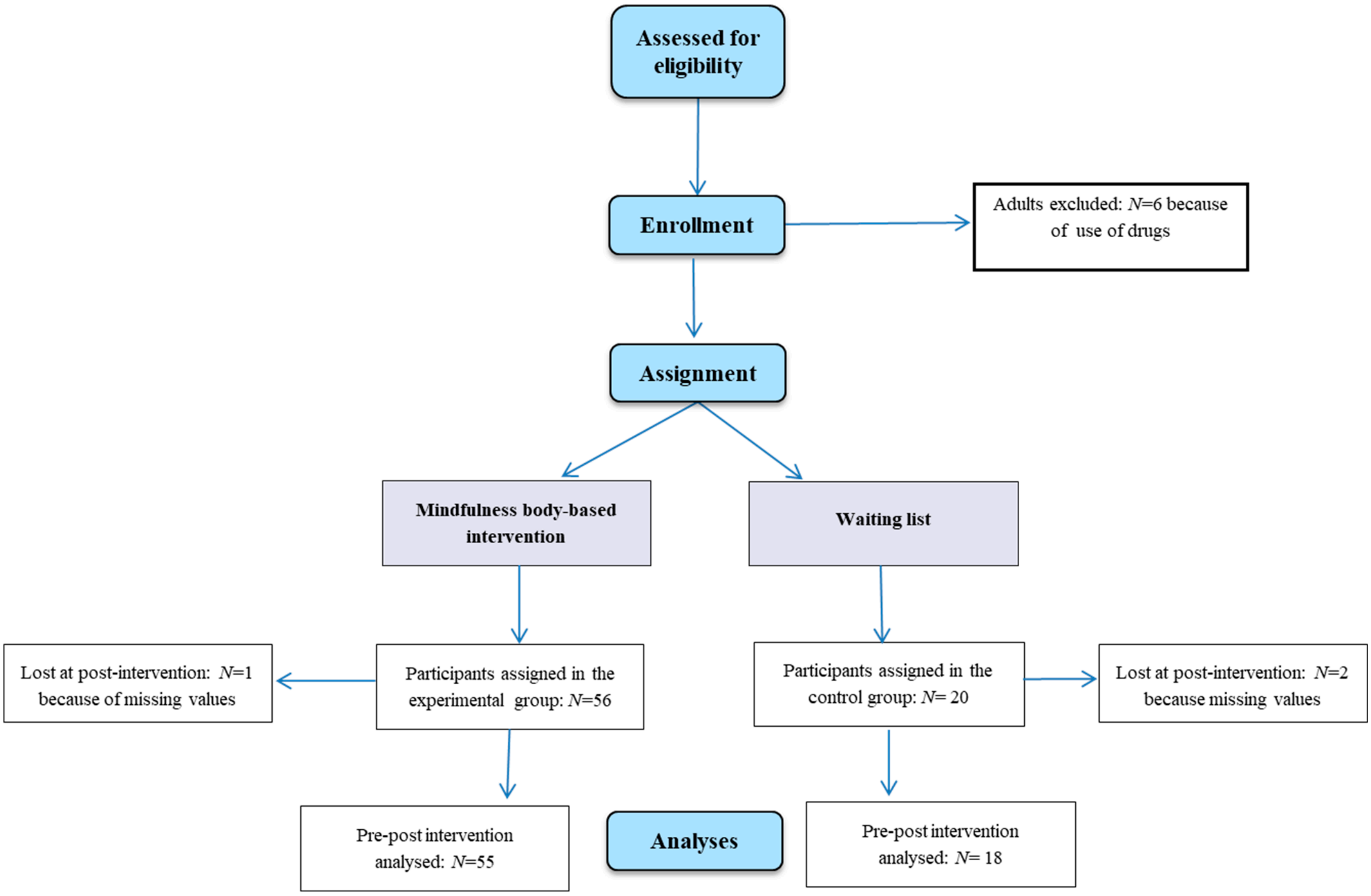

Figure S1. Flowchart of participants.

Table S1. The Mindfulness Body-based Intervention: procedure, aims, and target effects.

Session	*Procedure*	*Aims*	*Target effects*
1	***Introduction***: Watching videos and slides about Corso Base and global self-awareness. The path and the aims of the Corso Base are introduced. Overcoming any initial difficulties (restlessness, embarrassment, etc.) ***Preparation***: Guided meditation for interoceptive awareness. Feeling breath and bodily sensations in the present and then experience bodily changes remembering episodes from one's past. ***Emotional work***: To feel and express the emotions emerging by expressing one's needs. ***Being Mindful***: Guided meditation to perceive the self-integrity. ***Expression and sharing***: Social sharing of interoceptive experience and emotions. ***Mindful Meditation***: Evolution 5-phases active meditation aimed at rebalancing the sympathetic/parasympathetic system.	To become familiar with the first practices such as interoception, silence, sharing, circle time, role play, breathwork, etc.	To acquire mindful basic skills and understand the path and the aims of the Corso Base.
2	***Introduction***: Watching videos and slides about the importance of primary emotional, attunement and physical regulation with maternal figure and watch video about neuro-emotional systems (i.e., Schore's theory, Panksepp's theory). ***Preparation:*** Body scan from the head to the belly, perceiving the body parts where breath does not seem to flow easily due to some tensions or pains. Describing tensions, and giving them a shape, color or heat/cold sensation. Participants are invited to associate emotions to these bodily sensations. ***Guided meditation for interoceptive awareness:*** Feeling breath and bodily sensations in the present and then experience body changes while remembering past episodes about relationship with mother. ***Initial experiences*** in caring relationships, nurturing, tenderness, holding and handling. ***Emotional work***: Breathwork expressing emotions and needs to the *mother*. ***Being Mindful***: Guided meditation to perceive the self-integrity and interoceptive. ***Expression and sharing***: Social sharing of interoception and emotions during and after meditation. ***Mindful Meditation***: *Maternage* - static meditation, exploring the sensations of vulnerability and delicacy.	To explore the primary relationship with the maternal figure.	To acquire mindful basic skills and understand personal needs not satisfied in childhood by taking care of them in adulthood.

3	***Introduction***: Watching videos and slides about the importance of primary emotional and physical regulation with the father.<br>***Preparation***: Guided meditation for interoceptive awareness. Feeling breath and bodily sensations in the present and then perceiving bodily changes while remembering past episodes about relationship with father.<br>***Initial experiences*** in caring relationships, protection, holding and handling, physical play.<br>***Body scan*** from the head to the belly, feeling the bodily parts where breath does not seem to flow easily due to some tensions or pains. Describe tensions, give them a shape, color, or heat/cold sensation. Participants are invited to associate emotions to these bodily sensations.<br>***Emotional work***: Breathwork expressing emotions and needs to the *father*.<br>***Being Mindful***: Guided meditation to perceive the self-integrity and interoceptive awareness.<br>***Expression and sharing***: Social sharing of emotions during and after meditation.<br>***Mindful Meditation***: *Paternage* static meditation, exploring the sense of protection.	To explore the primary relationship with the father figure in a different way.	To acquire mindful basic skills and understand their own needs not satisfied in childhood by taking care of them in adulthood.
4	***Introduction***: Watching videos and slides about the importance of traumatic events in life and examples of their impact on well-being. Exercises and experiences are dedicated to the most challenging memories of life. This is possible by the confidence with the tools that each one has developed and thanks to the learning of a certain mindfulness ability that provides greater support to the participants.<br>***Preparation***: Guided meditation for interoceptive awareness. Feeling breath and bodily sensations in the present and then experience bodily changes remembering episodes from one's trauma in the past.<br>***Emotional work***: Breathwork expressing emotions and needs while remembering traumatic events.<br>***Role Play*** about traumatic events.<br>***Being Mindful***: guided meditation to perceive the self-integrity and interoceptive awareness.<br>***Expression and sharing***: social sharing of emotions.<br>***Mindful Meditation***: Evolution 5-phases active meditation aimed at rebalancing the sympathetic/parasympathetic system.<br>***Expression and sharing***: Circle Time with sharing.	To face and overcome traumatic events by regaining full awareness	Each emotional, bodily or cognitive experience is brought back to a lived experience of Self-integrity that allows the person's resources to be redirected in their expression of Self in their current life, processing difficult experiences that can condition the subject's way of living.
5	Seminar dedicated to mindfulness and interoception.<br>All time is spent in a sequence of different meditation techniques to develop one's fullness of awareness and well-being: static and dynamic meditations; use of sound, draw and meditation techniques.	To increase interoception and body awareness, as the ability to pay attention and to be aware of internal bodily sensations, emotions and feelings.	To grow one's authenticity and capacity to deep awareness.

6	***Introduction***: Watching videos and slides about the importance of interoception, and the perception of personal power, assertiveness and self-determination.<br>***Preparation***: Guided meditation for interoceptive awareness; feeling breath and bodily sensations in the present.<br>***Being Mindfull***: Grounding, relaxing, and activating exercises to stimulate parasympathetic and sympathetic systems, respectively.<br>***Identification***: Guided meditation for identifying the body parts that are relaxed or tense. Group exercises to develop feelings of personal power, such as saying one's name and identifying signature strengths.<br>***Role Play*** about personal power.<br>***Being mindful***: To allow thoughts and feelings to come and go, stay in silence, and be aware of the breath.<br>***Expression and sharing***: To share if one feels comfortable or not while experiencing bodily sensations during relaxing and activating exercises, and noticing the benefits of arousal modulation. Difficulties and strengths in expressing personal power.<br>***Mindful Meditation***: Three sounds exercise (i.e., producing sounds and paying attention to bodily sensations), guided meditation on the breath and its importance; allowing thoughts and feelings to come and go, stay in silence, and be aware of the breath. Psychosomatic drawing of what the participants feel and how they perceive their body during body scan.<br>***Expression and sharing***: Circle time with social sharing of interoception and emotions.	To rediscover personal strength and awareness.	To recognize how the bodily perception changes when it is associated with unpleasant experiences and know how to reconnect with a pleasant internal state as basis of individual well-being. To develop feelings of personal power.
7	***Introduction***: Watching videos and slides about the importance of interoception, the perception of the pleasantness of the body and the perception of the wholeness of oneself as the basis of self-integrity. To introduce the concept of eudaimonic well-being to reinforce personal growth, self-determination, and autonomy.<br>***Preparation***: Guided meditation for interoceptive awareness, feeling breath and bodily sensations in the present. To recognize how the bodily perception changes when it is associated with unpleasant experiences and knowing how to reconnect with a pleasant internal state as basis of individual well-being.<br>***Being Mindful***: Guided meditation to perceive the self-integrity and interoceptive awareness; to allow thoughts and feelings to come and go, stay in silence, and be aware of the breath.<br>***Expression and sharing***: Social sharing of mindfulness experience.<br>***Mindful Meditation***: Three sounds exercises; guided meditation on the breath and its importance, producing sounds and paying attention to bodily sensations. To recognize how the body perception changes when it is associated with unpleasant experiences and knowing how to reconnect with a pleasant internal state is seen as the basis of individual well-being. To allow thoughts and feelings to come and go, stay in silence, and be aware of the breath. Psychosomatic drawing of what the participants feel and how they perceive their body during body scan.<br>***Expression and sharing***: Social sharing of interoception and experienced emotions.	To develop emotional self-awareness and empathy. To deep the key elements of the concept of eudaimonic well-being.	To enhance the capacity to identify, express, and share pleasant and unpleasant emotions.

8	***Introduction***: Watching slides about the ability to suspend one's judgments and automatic reactions.<br>***Preparation***: Guided meditation for interoceptive awareness, feeling the breath and the bodily sensation in the present. To recognize how the bodily perception changes when we recall the subjective experiences in which we have received unpleasant judgments and injunctions and how this conditions our current experience.<br>***Emotional work***: Breathwork allows to express emotions and needs in the experience with unpleasant judgments and injunctions.<br>***Being aware***: Guided meditation to return to the integrity of the self and interoceptive awareness; letting thoughts and feelings come and go, being silent and being aware of the breath.<br>***Expression and sharing:*** Social sharing and circle time.<br>***Mindful meditation:*** Guided meditation on the breath and the Self, its importance. To recognize how the perception of the body changes in experiences with unpleasant judgments and injunctions. To let thoughts and feelings come and go, being silent and being aware of the breath that participants feel and how they perceive their body during the body scan.<br>***Expression and sharing***: Social sharing of interoceptive experience and emotions.	To develop the ability to suspend one's judgments and automatic reactions.	To make a direct and intuitive experience of the nature of reality, understanding how the received conditioning distances us from this goal. To understand that careful and non-judgmental observation of one's interoceptive, mental and emotional experiences in the present moment, including bodily sensations, emotions, thoughts and perceptions, is the basis of well-being.
9	***Introduction***: Watching videos and listening to music that reconnects to experiences of loss.<br>***Preparation***: guided meditation for interoceptive awareness, feel breath and bodily feeling in the present and then perceive bodily changes remembering episodes of losses.<br>***Emotional work***: Breathwork expressing emotions and needs in losses events.<br>***Being Mindful:*** Guided meditation to perceive the self-integrity and interoceptive awareness.<br>***Expression and sharing***: Social sharing of interoception and emotions.<br>***Preparation***: Exercise of *Being a Planet*- where each body part requires attention as in an ecosystem.<br>***Identification***: Guided meditation to develop a sense of interconnectedness with all living beings.<br>***Being Mindful***: To allow thoughts and feelings to come and go, stay in silence, and be aware of the breath.<br>***Expression and sharing***: Sharing one’s experience.	To deal with losses, to mourning to letting go of one's attachments.	To promote a sense of interconnectedness with other people and nature, expanding global and ecological awareness.
